# Factors Associated with Job Satisfaction in Medical Laboratory Professionals during the COVID-19 Pandemic: An Exploratory Study in Ontario, Canada

**DOI:** 10.3390/ejihpe13010004

**Published:** 2022-12-31

**Authors:** Joyce Lo, Yusra Fayyaz, Sharan Jaswal, Basem Gohar, Amin Yazdani, Vijay Kumar Chattu, Behdin Nowrouzi-Kia

**Affiliations:** 1 Department of Occupational Science and Occupational Therapy, Temerty Faculty of Medicine, University of Toronto, Toronto, ON M5G 1V7, Canada; 2 Department of Population Medicine, The University of Guelph, Guelph, ON N1G 2W1, Canada; 3 Centre for Research in Occupational Safety & Health, Laurentian University, Sudbury, ON P3E 2C6, Canada; 4 Canadian Institute for Safety, Wellness & Performance, School of Business, Conestoga College Institute of Technology and Advanced Learning, Kitchener, ON N2G 4M4, Canada; 5 Center for Transdisciplinary Research, Saveetha Dental College, Saveetha Institute of Medical and Technical Sciences, Saveetha University, Chennai 600077, India; 6 Department of Community Medicine, Faculty of Medicine, Datta Meghe Institute of Medical Sciences, Wardha 442107, India

**Keywords:** job satisfaction, medical laboratory technologists, medical laboratory technicians, COPSOQ III

## Abstract

Job satisfaction has been widely studied across several healthcare disciplines and is correlated with important outcomes such as job performance and employee mental health. However, there is limited research on job satisfaction among medical laboratory professionals (MLPs), a key healthcare group that aids in diagnosis, treatment, and patient care. The objective of this study is to examine the demographic and psychosocial factors associated with job satisfaction for MLPs in Ontario, Canada during the COVID-19 pandemic. A survey was administered to medical laboratory technologists (MLTs) and medical laboratory technicians/assistants (MLT/As) in Ontario, Canada. The survey included demographic questions and items from the Copenhagen Psychosocial Questionnaire, third edition. Binary logistic regressions were used to examine the association between job satisfaction and demographic variables and psychosocial work factors. There were 688 MLPs included in the analytic sample (72.12% response rate). Having a higher sense of community at work was correlated with higher job satisfaction in both MLT (OR = 2.22, 95% CI: 1.07–4.77) and MLT/A (OR = 3.85, 95% CI: 1.12–14.06). In addition, having higher stress was correlated with lower job satisfaction in both MLT (OR = 0.32, 95% CI: 0.18–0.57) and MLT/A (OR = 0.26, 95% CI: 0.10–0.66). This study provides preliminary evidence on factors associated with job satisfaction in MLT and MLT/A. The findings can be used to support organizational practices and policies to improve psychosocial work factors.

## 1. Introduction

The COVID-19 pandemic has revealed the vulnerability of medical laboratory professionals (MLPs) and the seriousness of MLP shortages in many jurisdictions, including Canada. This group includes medical laboratory technologists (MLTs) and medical laboratory technicians/assistants (MLT/As). In Ontario, MLTs and MLT/As are experiencing increased workloads because of the COVID-19 pandemic [1]. Additionally, in 2019, the Medical Laboratory Professionals’ Association of Ontario (MLPAO) indicated a significant labour shortage of MLTs in Ontario [2]. The increased workload exacerbates this situation and leads to increased work exhaustion, job dissatisfaction, and turnover rates [3]. Similarly, in a qualitative study that examined the stressors of MLPs during the pandemic, staff shortage was a profound contributor to work stress [4]. Notably, the results revealed that staff shortage was an area of concern well before the pandemic. In turn, this leads to poor mental health outcomes and stress leaves, creating a constant cycle of increased workload, poor mental health, staff illness and absences, and staff shortages [5].

The impact of job satisfaction has been widely studied across several healthcare professions, such as nurses [6,7], physicians [8,9], and social workers [10,11]. Job satisfaction can be broadly defined as the extent to which people like their jobs [12]. This multifaceted construct captures elements of satisfaction related to pay, work, supervision, professional opportunities and benefits, organizational practices, and relationships with co-workers [13]. The importance of job satisfaction has mainly been emphasized in the literature because of its positive impact on job performance [14,15] and correlations with employee withdrawal [16], employee mental health [17], and burnout [18]. Employees who are highly satisfied with their jobs are more productive [19] and stay longer with the organization [20]. Additionally, job satisfaction in healthcare professionals has been found to affect the quality of care and patient satisfaction [21].

Recent data and research on job satisfaction have mainly focused on physicians and nurses [22]. However, understanding job satisfaction among MLPs is equally important given their critical role in patient care, as they provide essential testing that aid in the detection, diagnostics, and treatment of diseases, facilitate efforts to monitor health, and engage in disease prevention [23].

A study by Garcia et al. [22] found that overall, MLPs reportedly have high job satisfaction levels. However, more than half of the respondents indicated that they were not adequately compensated. Furthermore, because of burnout, many MLPs working in rural areas considered changing careers completely. In addition, there were statistically significant differences in job satisfaction by age group and gender. Another factor related to ratings of job satisfaction is perceptions of how well hospitals and laboratory leadership responded to the COVID-19 pandemic [23].

### State of the Art

The literature suggests that numerous factors are associated with job satisfaction, such as job performance [14,15], employee mental health [17], and retention [20]. Notably, the impact of the COVID-19 pandemic of MLPs on job satisfaction remains poorly understood. Due to the stressors of the pandemic and increased burden among MLPs, we can expect job satisfaction to be negatively impacted. Given the importance of MLPs in the healthcare system and the impact of job satisfaction on various work outcomes, it is necessary to investigate job satisfaction in this group. Therfore, our study aimed to examine the factors associated with job satisfaction for MLPs during the COVID-19 pandemic in Ontario, Canada.

## 2. Materials and Methods

### 2.1. Procedure

This study is part of a larger study that included 929 MLT and 1866 MLT/A in Ontario, Canada. The current study utilized a cross-sectional design. A survey was administered via REDCap [24,25] for MLTs and MLT/As in Ontario. In the study, we used validated questionnaires, the survey included questions about MLT and MLT/A demographics, psychosocial work environment, occupational characteristics and mental health and well-being. The research project was approved by the research ethics board at the University of Toronto (REB#00039635). 

### 2.2. Participants

We partnered with the MLPAO, the provincial organization representing MLPs, who led the recruitment process and the dissemination of the questionnaire for this study. In September of 2020, MLTs and MLT/As were invited through an electronic letter stating the study’s objectives and respondents’ rights as research participants. The survey was also distributed through electronic letter. Two reminders were sent to those who did not respond. 

To participate, MLT and MLT/A had to meet the following eligibility criteria (1) Working as of March 11, 2020 (start of the COVID-19 pandemic), (2) Clinical practice location is Ontario, (3) Providing direct or indirect clinical patient care, (4) Actively registered with the College of MLT of Ontario (only required for MLT). There were 553 MLTs and 401 MLT/As who met the study’s eligibility criteria. A sample size calculation was done to determine the adequate sample size to detect small to moderate differences in the level of job satisfaction [26].

#### MLT versus MLT/A

MLTs are regulated health professionals while MLT/As are not regulated [27]. In Ontario, regulated health professions are governed under the Regulated Health Professions Act [28] and practice under a regulatory college (licencing board). Both MLTs and MLT/As can work in private and public sectors, including hospitals, research facilities, and educational institutions [27]. However, MLTs conduct medical laboratory tests to assist in the diagnosis, treatment and prevention of disease while MLT/As conduct routine medical laboratory tests and prepare medical laboratory equipment [29,30]. Medical laboratory technicians and medical laboratory assistants were grouped together for the analysis because they are categorized under the same title according to the National Occupational Classification [30]. 

### 2.3. Instruments

Our survey included demographic questions about the respondent’s age, gender, ethnicity, marital status, job classification, highest level of education, employment status, number of children living at home, and accommodations required at work due to disability. Disability was self-reported as a mental or physical health impairment that interferes with functioning.

We used the Copenhagen Psychosocial Questionnaire, third edition (COPSOQ III). The COPSOQ III helps measure psychosocial conditions and organizational development in the workplace [31]. The questionnaire uses 48 items from the COPSOQ III, medium version, assessing the following domains: Demands at Work, Work Organization and Job Contents, Interpersonal Relations and Leadership, Work–Individual Interface, Social Capital, Offensive Behaviors, and Health and Well-being [32]. Twenty-seven dimensions were assessed across these domains, including job satisfaction, social support, burnout, and work pace. Five-point Likert scale–type items are used and scaled to the interval of 0–100 (0, 25, 50, 75, & 100). For example, the question “Regarding your work in general. How pleased are you with your job as a whole, everything taken into consideration?” uses Very satisfied (100), Satisfied (75), Neither/Nor (50), Unsatisfied (25), Very unsatisfied (0). 

The COPSOQ III has been psychometrically assessed for validity and internal reliability in various countries, including Canada [32,33]. Additionally, it is valid and reliable across employee populations, industries, and different social contexts [32]. The COPSOQ III also has Canadian standardized data that was used to compare to our population [33]. In an international study evaluating the COPSOQ III psychometric properties, floor effects were present for dimensions such as Job Insecurity (19%). Furthermore, ceiling effects were seen for the following dimensions: Sense of Community at work (30%), Social Support from Colleagues (21%) and Supervisor (25%), Meaning of Work (25%) and Quality of Work (26%) [32]. 

The following question was used to examine the impact of COVID-19 on job satisfaction: “Since COVID-19, my current response is ___ before”, with the three response options ‘better than, ‘the same as’, and ‘worse than’. 

### 2.4. Data Analysis

All statistical analyses were performed using R studio version 2021.09.2 for macOS [34]. Demographic information was summarized using descriptive statistics (means and standard deviation for continuous variables, frequencies, and percentages for categorical variables). COPSOQ III responses were reported as ‘high’ or ‘low’, and job satisfaction (dependent variable) was categorized as “satisfied” or “unsatisfied” based on the median values of the distribution [35]. Binary logistic regressions were used to determine the association between job satisfaction and demographic factors (age, gender, ethnicity, marital status, education level, number of children living at home, employment status, accommodation required at work due to disability) and psychosocial factors (COPSOQ III domains). Alpha level was set to 0.05. Odds ratios (OR) and 95% confidence intervals (CI) were reported. Multicollinearity was assessed using the variance inflation factor to ensure that the independent variables are not intercorrelated. Since the results were not intercorrelated, we did not report the findings. The impact of the COVID-19 pandemic on job satisfaction was examined using chi-squared tests.

## 3. Results

### 3.1. Demographics

The survey presents data from 688 MLPs across Ontario, Canada. The response rate was 72.12% (688/954). The respondent demographic variables are summarized in Table 1. Most respondents were white and identified as a woman, with the mean age of 43 years (SD = 11.7). Additionally, most respondents were MLTs and worked full time with community college or university level education. Around 75% of respondents were married or in a committed relationship and about half (48%) of the respondents had children at home. Furthermore, around 3% of MLPs identified needing an accommodation at work due to disability.

The relationship between the demographic variables and job satisfaction are shown for MLT in Table 2 and for MLT/A in Table 3. In the adjusted model for MLT, accommodation required at work due to disability was significant (OR = 3.44, 95% CI: 1.17–10.11). MLTs that required an accommodation were 3.44 times more likely to report job satisfaction than those who did not require an accommodation. Additionally, number of children living at home was also significant (OR = 0.56, 95% CI: 0.35–0.9). In other words, MLTs with 1–2 children were about half as likely to report job satisfaction than MLTs with 0 children. In the unadjusted model for MLT/As, only employment status was significant (OR = 1.89, CI: 1.1–3.24).

### 3.2. COPSOQ III Dimensions

The relationship between psychosocial workplace factors (COPSOQ III dimensions) and job satisfaction for MLT is shown in Table 4, and for MLT/As in Table 5. The unadjusted logistic regression analysis revealed that 20 out of 22 psychosocial dimensions (except influence at work and job insecurity) were significantly associated with job satisfaction in MLTs. In the adjusted model, high meaning of work (OR = 2.40, 95% CI: 1.35–4.33), high recognition (OR = 2.40, 95% CI: 1.29–4.49), high sense of community at work (OR = 2.22, 95% CI: 1.07–4.77), and high stress (OR = 0.32, 95% CI: 0.18–0.57) remained significant for MLTs. In other words, for example, MLTs with high stress were about one third as likely to report job satisfaction than MLTs with low stress. 

The relationship between psychosocial workplace factors and job satisfaction for MLT/As is shown in Table 5. The unadjusted logistic regression analysis showed that 19 out of 22 psychosocial dimensions (except work pace, influence at work, job insecurity, and insecurity over working conditions) were significantly associated with job satisfaction in MLT/As. In the adjusted model, high social support from supervisor (OR = 2.49, 95% CI: 1.05–5.97), high sense of community at work (OR = 3.85, 95% CI: 1.12–14.06), high job insecurity (OR = 0.46, 95% CI: 0.21–0.98), high insecurity over working conditions (OR = 2.37, 95% CI: 1.09–5.37), high vertical trust (OR = 2.85, 95% CI: 1.18–7.03), and high stress (OR = 0.26, 95% CI: 0.10–0.66) remained significant for MLT/As. 

The impact of COVID-19 on job satisfaction is shown in a cross-tabulation in Table 6 for MLTs and MLT/As. More than half (50.9%) of the MLTs and 48.3% of MLT/As reported their job satisfaction being worse than the start of the pandemic (*p* < 0.001).

## 4. Discussion

We aimed to explore the demographic and psychosocial work factors associated with job satisfaction for MLTs and MLT/As in Ontario, Canada. The results revealed that most COPSOQ III dimensions were significantly associated with job satisfaction in MLTs and MLT/As in the unadjusted models. Furthermore, for both groups, influence at work was not statistically significant. In the adjusted model, having a high sense of community at work was associated with higher job satisfaction in both groups. In comparison, high stress was associated with lower job satisfaction in both groups. Meaning of work and recognition were associated with job satisfaction in MLTs while social support from supervisor, job insecurity, insecurity over working conditions and vertical trust were associated with job satisfaction in MLT/As.

The relationship between the COPSOQ III dimensions and the job satisfaction dimension has not been extensively studied. However, in a study by Hansson et al. [36], high levels of meaningfulness in work was associated with higher job satisfaction for midwives in Sweden. In the present study, having a high meaning of work score was also related to higher job satisfaction in the unadjusted model for both MLTs and MLT/As but remained significant for only MLTs in the adjusted model. Furthermore, the researchers also found that the following variables explained most of the variance of job satisfaction in this sample: possibilities for development, quality of work, role conflicts, burnout, and recognition [36]. Similar to the present study, all these dimensions were significantly associated with job satisfaction for both MLTs and MLT/As, but only recognition remained significant in the adjusted model for MLTs. Another study by Goetz et al. [37] used the COPSOQ to look at psychosocial factors and work-related outcomes for general medicine practice assistants in Germany. Similar to the current study, job satisfaction was associated with a higher of sense of community in practice assistants. The researchers found that job satisfaction was also associated with higher scores in meaning of work and role-clarity, and lower scores in quantitative demands and role conflict. To compare, having a high score in meaning of work was only significant for MLTs in the adjusted model. The researchers also found that quality of leadership had a notably strong association with job satisfaction. In the current study, quality of leadership was associated with job satisfaction for both MLTs and MLT/As but only in the unadjusted model.

Only two dimensions remained significant in the adjusted models for both groups: a sense of community at work and stress. The literature supports this finding in other healthcare groups. For example, one study found job satisfaction was higher for clinicians who reported a perceived higher accuracy of clinician-to-clinician communication [38]. The strength of accuracy in clinician-to-clinician communication is important for demonstrating a shared sense of understanding for optimal patient outcomes. Another study on psychiatric nurses found that nurse-physician collaboration was associated with higher job satisfaction [39]. In the present study, having a sense of community at work is measured by rating one’s atmosphere between themselves and colleagues. A positive work atmosphere between colleagues may result in higher job satisfaction because of the established respect and trust. The findings also support the literature on the relationship between stress and job satisfaction. For example, several studies have found that stress is a strong predictor of job satisfaction in healthcare workers [40,41,42]. High stress may be correlated with lower job satisfaction because conflict and strain can impact individual well-being. The relationship between stress and job satisfaction in this group may also be explained by the significant labour shortage of MLPs in Ontario, as increased workloads negatively impact stress and job satisfaction [1,3,4].

The majority of MLTs and MLT/As reported their job satisfaction as worse compared to before the COVID-19 pandemic. The impact of the COVID-19 pandemic on job satisfaction could be due to the notable staffing shortages, lack of recognition, and poor working environments, as reported in a qualitative study by Gohar and Nowrouzi-Kia [4]. Aligned with our findings, a study looking at MLPs in the United States reported that job dissatisfaction may have doubled during the first months of the pandemic [23]. Furthermore, the quality of response to COVID-19 from administration was a significant factor influencing job satisfaction. Job satisfaction has been reported to be a protective predictor for burnout [3]. As the prevalence of burnout in MLPs during COVID-19 was 72.3%, this supports our finding that MLPs are experiencing higher job dissatisfaction compared to before the COVID-19 pandemic. 

### Strengths and Limitations

To our knowledge, this is the first study to use the COPSOQ factors to look at job satisfaction in MLTs and MLT/As. One strength of this study is the use of the COPSOQ III, which has been assessed for validity and reliability in various countries and employee populations. Using a validated measure, the findings can be interpreted with international data. While this study provides insight into understanding job satisfaction in this essential healthcare group, there are some limitations. First, because this is a cross-sectional study, this limits the opportunity to establish casual conclusions. Second, although we had the support of the MLPAO for recruitment, not all MLTs in Ontario were included because MLPAO membership is voluntary. Thus, limiting the generalizability of our results for MLTs in Ontario. Lastly, dichotomizing the COPSOQ III variables leads to the loss of information. Although, the dichotomization is based on the median values of the distribution, this still presents limitations on the statistical power.

## 5. Conclusions

This study provides preliminary evidence of demographic and psychosocial factors associated with job satisfaction in MLPs during COVID-19. The findings can be applied to help develop future interventions or studies to investigate if job satisfaction can be improved. For example, based on the results, future interventions should focus on reducing stress and promoting a sense of community to improve job satisfaction in MLP workplaces. Moreover, health professional organizations, such as the MLPAO, can disseminate the findings to MLP workplaces and provincial parliament members to develop workplace policies, services or practices that target psychosocial risk factors. Future studies should continue to look at job satisfaction in MLPs. For example, conducting a cohort study can address the limitations of using a cross-sectional design and allow for exploring casual relationships between job satisfaction in MLPs and psychosocial work factors.

## Figures and Tables

**Table 1:** Demographic information of MLT and MLT/A respondents.

	Frequency	Percentage
**Job classification (*n* = 688)**		
Medical Lab Technologist	440	64
Medical Lab Technician	187	27.2
Medical Lab Assistant	61	8.9
**Gender (*n* = 687)**		
Woman	627	91.3
Man	57	8.3
Other	3	0.4
**Age (*n* = 649)**		
21–32	156	24
33–42	164	25.3
43–52	162	25
53–76	167	25.7
**Ethnicity (*n* = 704)**		
Caucasian/White	565	80.3
Other	139	19.7
**Marital Status (*n* = 686)**		
Single	112	16.3
Married/Common Law/Committed Relationship	516	75.2
Separated/Divorced	52	7.6
Widowed	6	0.9
**Highest level of education attained (*n* = 725)**		
High school or less	20	2.8
Vocational school or Community College	426	58.8
University	279	38.5
**Number of children living at home (*n* = 674)**		
0	355	52.7
1–2	271	40.2
3–5	48	7.1
**Employment status (*n* = 733)**		
Full-time	523	71.4
Part-time	167	22.8
Other	43	5.9
**Accommodation required at work** **due to disability (*n* = 687)**		
Yes	24	3.5
No	640	93.2
Choose not to answer	23	3.3

**Table 2:** Demographic factors associated with job satisfaction in MLT.

	Job Satisfaction *n* (%) Satisfied Unsatisfied		Unadjusted Odds Ratio Estimate	95% CI	Adjusted Odds Ratio Estimate	95% CI
**Gender**						
Woman	226 (56.9)	171 (43.1)	1			
Man	24 (58.5)	17 (41.5)	0.94	0.49–1.8	1.16	0.57–2.35
**Age group**						
21–32	55 (59.8)	37 (40.2)	1			
33–42	46 (48.4)	49 (51.6)	1.58	0.88–2.84	1.71	0.94–3.1
43–52	53 (57.6)	39 (42.4)	1.09	0.61–1.97	1.28	0.65–2.52
53–76	86 (63.7)	49 (36.3)	0.85	0.49–1.47	0.93	0.49–1.78
**Ethnicity**						
Caucasian/White	220 (57.9)	160 (42.1)	1			
European	14 (70)	6 (30)	0.59	0.22–1.57	0.76	0.25–2.31
Asian	23 (60.5)	15 (39.5)	0.9	0.45–1.77	0.6	0.13–2.77
Other	9 (50%)	9 (50)	1.38	0.53–3.54	0.65	0.14–3.12
**Marital Status**						
Single	35 (60.3)	23 (39.7)	1			
Married/Common Law/Committed Relationship	191 (55.2)	155 (44.8)	1.23	0.70–2.20	1.64	0.86–3.11
Separated/Divorced	22 (71)	9 (29)	0.63	0.24–1.59	0.96	0.34–2.72
Widowed	4 (80)	1 (20)	0.42	0.01–3.29	0.27	0.03–2.87
**Highest level of education attained**						
University	126 (54.5)	105 (45.5)	1			
High school or less	4 (57.1)	3 (42.9)	0.90	0.20–4.11	0.61	0.091–4.1
Vocational school or Community College	21 (58.3)	15 (41.7)	0.86	0.42–1.75	2.32	1.02–5.25
**Number of children living at home**						
0	128 (54)	109 (46)	1			
1–2	106 (59.9)	71 (40.1)	0.79	0.53–1.17	0.56 *	0.35–0.9
3–5	14 (66.7)	7 (33.3)	0.59	0.22–1.5	0.37	0.13–1.08
**Employment status**						
Full time	202 (56.6)	153 (43.4)	1			
Part time	50 (60.2)	33 (39.8)	0.87	0.53–1.42	0.82	0.28–2.32
Other	13 (56.5)	10 (43.5)	1.02	0.43–2.38	0.75	0.28–2.6
**Accommodation required at work due to disability**						
No	242 (59.2)	167 (40.8)	1			
Yes	6 (31.6)	13 (68.4)	3.14 *	1.17–8.43	3.44 *	1.17–10.11

Note. * *p* < 0.05.

**Table 3:** Demographic factors associated with job satisfaction in MLT/A.

	Job Satisfaction *n* (%) Satisfied Unsatisfied		Unadjusted Odds Ratio Estimate	95% CI	Adjusted Odds Ratio Estimate	95% CI
**Gender**						
Men	8 (50)	8 (50)	1			
Women	124 (53.9)	106 (46.1)	0.85	0.31–2.35	0.51	0.13–1.82
**Age group**						
21–32	34 (53.1)	30 (46.9)	1			
33–42	34 (49.3)	35 (50.7)	1.17	0.59–2.3	1.08	0.49–2.39
43–52	40 (56.3)	31 (43.7)	0.88	0.45–1.73	0.82	0.37–1.81
53–76	18 (58.1)	13 (41.9)	0.81	0.34–1.95	0.69	0.26–1.86
**Ethnicity**						
Asian	20 (54.1)	17 (45.9)	1			
Caucasian/White	100 (54.1)	85 (45.9)	1.00	0.49–2.03	0.46	0.09–2.35
European	3 (30)	7 (70)	2.75	0.61–12.29	1.53	0.21–10.94
Other	14 (63.6)	8 (36.4)	0.67	0.23–1.99	0.37	0.07–2.04
**Marital Status**						
Single	29 (53.7)	25 (46.3)	1			
Married/Common Law/Committed Relationship	87 (51.2)	83 (48.8)	1.11	0.6–2.04	0.87	0.41–1.83
Separated/Divorced	15 (71.4)	6 (28.6)	0.46	0.16–1.38	0.38	0.11–1.29
**Highest level of education attained**						
Vocational school or Community College	112 (53.1)	99 (46.9)	1			
University	28 (53.8)	24 (46.2)	0.97	0.52–1.79	1.01	0.17–6.08
High school or less	10 (66.7)	5 (33.3)	0.58	0.17–1.70	0.51	0.12–2.13
**Number of children living at home**						
0	63 (53.4)	55 (46.6)	1			
1–2	46 (48.9)	48 (51.1)	1.19	0.69–2.06	1.09	0.58–2.05
3–5	19 (70.4)	8 (29.6)	0.49	0.19–1.18	0.4	0.14–1.14
**Employment status**						
Part time	54 (62.9)	30 (36.1)	1			
Full time	82 (48.8)	86 (51.2)	1.89 *	1.1–3.24	0.56	0.17–1.9
Other	13 (56.5)	10 (43.5)	1.38	0.54–3.54	0.31	0.1–1.65
**Accommodation required at work due to disability**						
No	129 (55.8)	102 (44.2)	1			
Yes	3 (60)	2 (40)	0.84	0.14–5.14	0.6	0.083–4.34

Note. * *p* < 0.05.

**Table 4:** Psychosocial factors associated with job satisfaction in MLT.

	Job Satisfaction *n* (%) Satisfied Unsatisfied		Unadjusted Odds Ratio Estimate	95% CI	Adjusted Odds Ratio Estimate	95% CI
**Quantitative demands**						
Low	95	79	1		1	
High	87	161	0.45 *	0.30–0.67	0.71	0.41–1.22
**Work pace**						
Low	113	92	1		1	
High	69	148	0.38 *	0.25–0.56	0.64	0.37–1.12
**Emotional demands**						
Low	105	73	1		1	
High	77	167	0.32 *	0.21–0.48	0.89	0.50–1.61
**Influence at work**						
Low	46	77	1		1	
High	136	163	1.40	0.91–2.16	0.59	0.312–1.11
**Possibilities for development**						
Low	61	134	1		1	
High	121	106	2.51 *	1.69–3.75	1.52	0.87–2.65
**Meaning of work**						
Low	46	117	1		1	
High	136	123	2.81 *	1.86–4.31	2.40 *	1.36–4.33
**Predictability**						
Low	29	110	1		1	
High	153	130	4.46 *	2.82–7.25	1.10	0.55–2.19
**Recognition**						
Low	35	141	1		1	
High	147	99	5.98 *	3.85–9.48	2.40 *	1.29–4.49
**Role clarity**						
Low	22	80	1		1	
High	160	160	3.64 *	2.20–6.24	1.78	0.89–3.62
**Role conflicts**						
Low	101	61	1		1	
High	81	179	0.27 *	0.18–0.41	0.63	0.36–1.13
**Quality of leadership**						
Low	33	128	1		1	
High	149	112	5.16 *	3.31–8.22	1.70	0.85–3.41
**Social support from colleagues**						
Low	17	49	1		1	
High	165	191	2.49 *	1.41–4.60	0.70	0.31–1.63
**Social support from supervisor**						
Low	51	137	1		1	
High	131	103	3.42 *	2.27–5.19	1.28	0.68–2.39
**Sense of community at work**						
Low	20	52	1		1	
High	162	188	2.24 *	1.30–3.99	2.22 *	1.07–4.77
**Job insecurity**						
Low	81	95	1		1	
High	101	145	0.82	0.55–1.21	1.37	0.80–2.38
**Insecurity over working conditions**						
Low	96	89	1		1	
High	86	151	0.53 *	0.36–0.78	0.97	0.57–1.68
**Vertical trust**						
Low	32	127	1		1	
High	150	113	5.27 *	3.37–8.43	1.89	0.97–3.71
**Organizational justice**						
Low	38	131	1		1	
High	144	109	4.55 *	2.96–7.13	1.28	0.67–2.43
**Work–life conflict**						
Low	96	82	1		1	
High	86	158	0.47 *	0.31–0.69	0.83	0.46–1.48
**Self-rated health**						
Low	64	142	1		1	
High	118	98	2.67 *	1.80–4.00	1.53	0.90–2.60
**Burnout**						
Low	89	45	1		1	
High	93	195	0.24 *	0.16–0.37	0.74	0.39–1.41
**Stress**						
Low	130	68	1		1	
High	52	172	0.16 *	0.10–0.24	0.32 *	0.18–0.57

Note: * *p* < 0.05.

**Table 5:** Psychosocial factors associated with job satisfaction in MLT/A.

	Job Satisfaction *n* (%) Satisfied Unsatisfied		Unadjusted Odds Ratio Estimate	95% CI	Adjusted Odds Ratio Estimate	95% CI
**Quantitative demands**						
Low	50	32	1		1	
High	58	90	0.41 *	0.24–0.71	0.74	0.32–1.70
**Work pace**						
Low	44	39	1		1	
High	64	83	0.68	0.40–1.17	1.36	0.57–3.32
**Emotional demands**						
Low	44	19	1		1	
High	64	103	0.27 *	0.14–0.49	0.61	0.26–1.44
**Influence at work**						
Low	42	60	1		1	
High	66	62	1.52	0.90–2.58	1.97	0.88–4.40
**Possibilities for development**						
Low	24	59	1		1	
High	84	63	3.28 *	1.86–5.91	1.52	0.62–3.74
**Meaning of work**						
Low	25	57	1		1	
High	83	65	2.91 *	1.66–5.22	1.55	0.69–3.53
**Predictability**						
Low	21	52	1		1	
High	87	70	3.08 *	1.72–5.68	0.59	0.22–1.53
**Recognition**						
Low	17	64	1		1	
High	91	58	5.91 *	3.21–11.34	1.70	0.63–4.63
**Role clarity**						
Low	17	58	1		1	
High	91	64	4.85 *	2.64–9.31	2.38	0.92–6.40
**Role conflicts**						
Low	38	27	1		1	
High	70	95	0.52 *	0.29–0.93	0.52	0.21–1.28
**Quality of leadership**						
Low	31	63	1		1	
High	77	59	2.65 *	1.54–4.63	0.72	0.30–1.68
**Social support from colleagues**						
Low	12	37	1		1	
High	96	85	3.48 *	1.75–7.37	1.11	0.36–3.49
**Social support from supervisor**						
Low	28	73	1		1	
High	80	49	4.26 *	2.45–7.56	2.49 *	1.05–5.97
**Sense of community at work**						
Low	9	30	1		1	
High	99	92	3.59 *	1.68–8.39	3.85 *	1.12–14.06
**Job insecurity**						
Low	56	51	1		1	
High	52	71	0.67	0.40–1.12	0.46 *	0.21–0.98
**Insecurity over working conditions**						
Low	35	39	1		1	
High	73	83	0.98	0.56–1.71	2.37 *	1.09–5.37
**Vertical trust**						
Low	23	70	1		1	
High	85	52	4.98 *	2.81–9.06	2.85 *	1.18–7.03
**Organizational justice**						
Low	18	53	1		1	
High	90	69	3.84 *	2.10–7.29	0.83	0.30–2.28
**Work–life conflict**						
Low	54	44	1		1	
High	54	78	0.56 *	0.33–0.95	1.58	0.68–3.76
**Self-rated health**						
Low	32	73	1		1	
High	76	49	3.54 *	2.06–6.19	2.09	0.97–4.57
**Burnout**						
Low	58	28	1		1	
High	50	94	0.26 *	0.14–0.45	0.78	0.29–2.11
**Stress**						
Low	79	36	1		1	
High	29	86	0.15 *	0.09–0.27	0.26 *	0.10–0.66

Note: * *p* < 0.05.

**Table 6:** Cross-tabulation of job satisfaction scores and COVID-19 scores.

MLT	N = 440	Better than *n* = 12 (2.70%)	The same as *n* = 204 (46.4%)	Worse than *n* = 224 (50.9%)	*p* < 0.001
Job satisfaction	Low	1	47	140	
	High	11	157	84	
MLT/A	N = 240	Better than *n* = 14 (5.83%)	The same as *n* = 110 (45.83%)	Worse than *n* = 116 (48.33%)	*p* < 0.001
Job satisfaction	Low	1	31	79	
	High	13	79	37	

## Data Availability

The data that supports the findings of this study is available from the corresponding author upon reasonable request.
